# Knowledge and Acceptability of Pap Smears, Self-Sampling and HPV Vaccination among Adult Women in Kenya

**DOI:** 10.1371/journal.pone.0040766

**Published:** 2012-07-10

**Authors:** Anne F. Rositch, Ann Gatuguta, Robert Y. Choi, Brandon L. Guthrie, Romel D. Mackelprang, Rose Bosire, Lucy Manyara, James N. Kiarie, Jennifer S. Smith, Carey Farquhar

**Affiliations:** 1 Department of Epidemiology, Johns Hopkins Bloomberg School of Public Health, Baltimore, Maryland, United States of America; 2 Department of Community Health, Kenyatta University, Nairobi, Kenya; 3 Department of Medicine, University of Washington, Seattle, Washington, United States of America; 4 Department of Epidemiology, University of Washington, Seattle, Washington, United States of America; 5 Department of Global Health, University of Washington, Seattle, Washington, United States of America; 6 Centre for Public Health Research, Kenya Medical Research Institute, Nairobi, Kenya; 7 Kenya Medical Training College, Nairobi, Kenya; 8 Department of Obstetrics and Gynecology, University of Nairobi, Nairobi, Kenya; 9 Department of Epidemiology, University of North Carolina, Chapel Hill, North Carolina, United States of America; IPO, Inst Port Oncology, Portugal

## Abstract

**Objectives:**

Our study aimed to assess adult women’s knowledge of human papillomavirus (HPV) and cervical cancer, and characterize their attitudes towards potential screening and prevention strategies.

**Methods:**

Women were participants of an HIV-discordant couples cohort in Nairobi, Kenya. An interviewer-administered questionnaire was used to obtain information on sociodemographic status, and sexual and medical history at baseline and on knowledge and attitudes towards Pap smears, self-sampling, and HPV vaccination at study exit.

**Results:**

Only 14% of the 409 women (67% HIV-positive; median age 29 years) had ever had a Pap smear prior to study enrollment and very few women had ever heard of HPV (18%). Although most women knew that Pap smears detect cervical cancer (69%), very few knew that routine Pap screening is the main way to prevent ICC (18%). Most women reported a high level of cultural acceptability for Pap smear screening and a low level of physical discomfort during Pap smear collection. In addition, over 80% of women reported that they would feel comfortable using a self-sampling device (82%) and would prefer at-home sample collection (84%). Nearly all women (94%) reported willingness to be vaccinated to prevent cervical cancer if offered at no or low cost.

**Conclusions:**

These findings highlight the need to educate women on routine use of Pap smears in the prevention of cervical cancer and demonstrate that vaccination and self-sampling would be acceptable modalities for cervical cancer prevention and screening.

## Introduction

Cervical cancer is a preventable disease, yet the number of cases globally is expected to almost double by the year 2025 [1]. Infection with high-risk genotypes of human papillomavirus (HR-HPV) is the primary cause of invasive cervical cancer; over 70% of all cervical cancers are attributable to infection with HPV-16 and 18 [2], [3]. Cervical cancer is the third most common cancer among women worldwide with an estimated 529,000 new cases in 2008, 85% of which occur in developing countries [4]. In 2008, WHO estimated that cervical cancer was the second most common cancer among Kenyan women [1], yet screening coverage is currently very low, according to the Kenyan national cervical cancer prevention strategic plan for 2012–2015 released in January of 2012 [5].

Traditionally, Pap smear, combined with treatment of cervical precancer and early stage cancer, has been successful in preventing up to 80% of invasive cervical cancer cases in developed countries [6], [7], [8]. In developing countries, however, high rates of cervical cancer mortality persist due to lack of effective screening programs and low uptake of Pap smear testing [9]. Reasons cited for the low uptake of screening include lack of awareness, inadequate access, exam discomfort, fear of finding cancer and logistical issues associated with obtaining screening [10], [11]. Newer technologies such as the careHPV DNA test (QIAGEN, Gaithersburg, MD, USA), cervico-vaginal self-sampling, and HPV vaccination have the potential to increase screening and reduce cervical cancer in developing countries [12], [13], [14].

Uptake of self-sampling has been shown to be successful and HPV testing from self-collected samples is highly sensitive for detection of cervical intraepithelial neoplasia grade 2/3 in both clinic and home settings [15], [16], [17]. Previous studies have shown that African women find self-sampling acceptable; for example, 80% of Ugandan women were willing to collect their own samples at home [18]. However, knowledge of new screening and cervical cancer prevention technologies remains low among most women, with studies documenting almost no awareness of HPV infection, HPV screening for women 30 years and older and adolescent vaccination for the prevention of future disease [10], [19], [20], [21], [22], [23], [24]. Awareness is even low among healthcare workers who are expected to provide preventative health services [25], [26], [27].

With the increase in technology and opportunity to prevent cervical cancer worldwide, it is important to understand attitudes and barriers of screening among women at high-risk of invasive cervical cancer in low-resource settings. Therefore, our study aimed to assess women’s knowledge of HPV and cervical cancer, and their attitudes towards potential screening strategies, including routine Pap smears, self-sampling for HPV DNA testing, and HPV vaccination. These data will be critical for successful implementation and high uptake of community-level cervical cancer screening programs.

## Methods

### Study Population and Design

Women were recruited into a cohort of HIV-1-discordant couples identified in voluntary counseling and testing centers in Nairobi, Kenya, from May 2007 to October 2009. Couples were eligible to enroll in the study if they reported ≥3 sex acts in the previous 3 months, planned to stay in Nairobi in their current relationship for at least 2 years, and if one member of the couple was HIV-1-infected and the other HIV-1 susceptible. Women who were pregnant and participants using antiretroviral therapy at the time of enrollment were excluded. At enrollment and at quarterly follow-up visits, the HIV status of the uninfected partner was determined using the Determine® HIV–1/2 Rapid Test (Abbott Laboratories, Abbott City, IL, USA) or Bioline Recombigen HIV Test (Standard Diagnostics, Suwon City, Korea), with confirmation by the Vironostika® HIV Uni-form II Ag/Ab ELISA kit (bioMérieux Inc., Durham, NC, USA). In the HIV-1-infected partner, CD4+ T-cell counts were taken at enrollment and every six months using a FACSCaliber flow cytometer (BD Biosciences, Franklin Lakes, NJ, USA).

At enrollment, clinical staff administered a questionnaire to obtain information on sociodemographic characteristics, sexual history and behavior, history of Pap screening, and medical history. Questionnaires were presented in English or Kiswahili, depending on participant preference, and were administered individually to ensure confidentiality. A medical examination was also conducted during biannual study visits, which included collection of a cervical Pap smear. All participants with abnormal cervical cytology were followed up with a repeat Pap test or colposcopy and biopsy as recommended.

### Assessment of Women’s Knowledge and Beliefs Regarding Cervical Cancer

At the final study visit, up to two years after study enrollment, clinical staff administered an extended questionnaire to obtain additional information on male circumcision, pregnancy history, domestic violence, ARV medication history, and cervical cancer and screening. Relevant questions fell into three categories to assess women’s: 1) knowledge of HPV and the causes and prevention of cervical cancer, 2) attitudes towards Pap smear screening after having had biannual screening in the study, and 3) attitudes towards routine Pap smear screening, self-sampling and HPV vaccination. Specifically, participants were asked if they had ever heard of HPV and if they knew the cause of cervical cancer or how to prevent it. Participants gave detailed histories of Pap smear screening prior to the study or the reasons why they had never had any screening, and were also asked about future intentions for Pap smear screening. Women rated their level of pain during study-conducted Pap smears, beliefs of cultural acceptability, and feelings of necessity of Pap smear screening. Finally, women were asked if they would feel comfortable collecting their own vaginal samples at home and if they would consider HPV vaccination if it were offered at no or low cost. No intervention or education on cervical cancer and screening was provided during the study unless medically indicated, and all responses were based on women’s one-time self-report.

Written informed consent was obtained from all participants. The study was conducted according to procedures approved by the University of Washington Institutional Review Board and the Kenyatta National Hospital Ethics and Research Committee.

### Statistical Analysis

A descriptive summary of knowledge and acceptability overall and stratified by HIV-status is presented. The numbers and percentages of each response are presented. Measures of Pap smear acceptability, necessity and pain-level are based on a scale of 0 to 100 and women’s responses are summarized by the mean, median and interquartile range. Exact logistic regression was used to calculate unadjusted odds ratios and 95% confidence intervals to describe the associations between baseline socioeconomic indicators and medical and sexual history and: 1) having ever had a Pap smear prior to study enrollment, 2) having ever heard of human papillomavirus, 3) knowing that Pap smear screening is conducted to prevent invasive cervical cancer, and 4) feeling that Pap smears are completely acceptable (rating of 100) versus less than completely acceptable. All analyses were conducted using SAS version 9.2 (SAS, Cary, NC, USA).

## Results

### Study Population

We interviewed 409 women from HIV-1-discordant couples, 268 (65%) of whom were HIV-positive at baseline with a median CD4+ T-cell count of 463 (interquartile range [IQR] 311–681; Table 1). The median age in the study population was 29 years (IQR 25–34), and the majority of women (97%) were married and had completed at least primary education (8 years). HIV-negative and positive participants were very similar, although HIV-negative women were slightly more likely to be married (99% vs. 95%), earn an income (35% vs. 28%), have never smoked (94% vs. 92%), be on hormonal contraceptive (24% vs.16%) and have fewer lifetime sexual partners (median 2 vs. 3) compared to HIV-positive women.

**Table 1:** Baseline characteristics of 409 HIV-negative and HIV-positive adult women.

	HIV-negative (N = 141) HIV-positive (N = 268) All women (N = 409)
	n (%) or median (IQR)		
**Age**	29 (26–35)	28 (24–34)	29 (25–34)
**Married**	139 (99%)	256 (96%)	395 (97%)
**Years of education**	8 (7–12)	8 (7–12)	8 (7–12)
**Earn monthly income**	49 (35%)	76 (28%)	125 (31%)
**History of smoking** 1			
Never	133 (94%)	246 (92%)	379 (93%)
Current	1 (1%)	4 (2%)	5 (1%)
Past	7 (5%)	18 (7%)	25 (6%)
**Number of live births**	2 (1–4)	2 (1–3)	2 (1–3)
**Abnormal Pap smear**	26 (19%)	62 (25%)	88 (23%)
**Current hormonal contraception use** 2	33 (24%)	44 (16%)	77 (19%)
**Age at sexual debut**	18 (16–20)	18 (16–19)	18 (16–19)
**Lifetime number of sex partners**	2 (2–3)	3 (2–4)	3 (2–4)
**Condom use at last sex**	111 (95%)	227 (94%)	338 (94%)
**CD4 T-cell count** 3	–	463 (311–681)	–

1 History of smoking: ≥1 cig/day for ≥6 consecutive months.

2 Hormonal contraception includes injection, oral and implant-based contraceptive.

3 Evaluated among HIV-1 infected female participants (n = 268).

Missing data: Abnormal Pap smear at baseline (n = 25); Hormonal contraception (n = 1); Median age at sexual debut (n = 1); Lifetime number of partners (n = 1); Condom use at last sex (n = 51); CD4 T cell (n = 17).

Abbreviations: HIV (human immunodeficiency virus); N (number); IQR (interquartile range); CD4 T-cell (T helper cells with cluster of differentiation 4 receptor).

### Knowledge and Beliefs Regarding Cervical Cancer

We assessed knowledge of cervical cancer, HPV, and screening among both HIV-negative and HIV-positive women (Table 2). HIV-positive women tended to be more aware that “HPV, a virus, or a sexually transmitted infection” causes cervical cancer compared to HIV-negative women (24% vs. 18%). While a substantial number of women did not know the cause of cervical cancer (78%), most women (69%) knew that Pap smears are used to test for cervical cancer. However, 82% of women did not know that Pap smear screening is an important part of preventing, not just detecting, cervical cancer. Nearly a quarter of women cited condom use and 13% reported being faithful to their partners as ways in which cervical cancer can be prevented. Only 18% of women had ever heard of HPV, and of these, 64% knew that HPV is transmitted through sexual intercourse and 35% did not know any mode of HPV transmission.

**Table 2:** HPV, Pap, and screening knowledge and acceptability by HIV infection status among adult women^1^.

	HIV-negative (n = 141)	HIV-positive (n = 268)	All women (n = 409)
	N (%)	N (%)	N (%)
**Reasons why Pap smear screening is conducted**			
To test for STIs	24 (17%)	36 (13%)	60 (15%)
To test for cervical cancer	100 (71%)	184 (69%)	284 (69%)
To test for other cancers	0 (0%)	6 (2%)	6 (1%)
To determine pregnancy	0 (0%)	0 (0%)	0 (0%)
Don’t know	23 (16%)	52 (19%)	76 (19%)
**Causes of cervical cancer**			
Smoking	1 (1%)	3 (1%)	4 (1%)
Poor sanitation/hygiene	0 (0%)	5 (2%)	5 (1%)
Multiple sex partners	18 (13%)	31 (12%)	49 (12%)
Pregnancy-related	1 (1%)	2 (1%)	3 (1%)
An STI/virus/HPV	26 (18%)	63 (24%)	89 (22%)
Exposure to pollution	8 (6%)	6 (2%)	14 (3%)
Family planning methods	0 (0%)	7 (3%)	7 (2%)
HIV/immunosuppression	0 (0%)	3 (1%)	3 (1%)
Other^4^	17 (12%)	11 (4%)	28 (7%)
Don’t know	72 (51%)	141 (53%)	213 (52%)
**Ways in which cervical cancer can be prevented**			
Use condoms	28 (20%)	59 (22%)	87 (21%)
Be faithful to your partner	19 (13%)	34 (13%)	53 (13%)
Have Pap smear screening	28 (20%)	46 (17%)	74 (18%)
Proper hygiene and washing	5 (4%)	15 (6%)	20 (5%)
Get a vaccine	7 (5%)	9 (3%)	16 (4%)
Can’t prevent	2 (1%)	3 (1%)	5 (1%)
Other^5^	6 (4%)	15 (6%)	21 (5%)
Not known	50 (35%)	104 (39%)	154 (38%)
**Ever heard of HPV**	28 (20%)	47 (18%)	75 (18%)
**If yes, ways in which HPV is transmitted** 2			
Sexual intercourse	18 (64%)	30 (64%)	48 (64%)
Touching infected person	1 (4%)	0 (0%)	1 (1%)
Coughing	0 (0%)	0 (0%)	0 (0%)
Contaminated food	0 (0%)	0 (0%)	0 (0%)
Not Known	9 (32%)	17 (36%)	26 (35%)
**Ever Pap smear prior to study enrollment**	27 (19%)	30 (11%)	57 (14%)
**If yes, where** 2			
Emergency room	1 (4%)	0 (0%)	1 (2%)
Private doctor	3 (11%)	1 (3%)	4 (7%)
Prenatal care	1 (4%)	1 (3%)	2 (4%)
Family planning clinic	5 (19%)	3 (10%)	8 (14%)
Hospital	12 (44%)	12 (40%)	24 (42%)
Research study	2 (7%)	11 (37%)	13 (23%)
Screening campaign/program	3 (11%)	0 (0%)	3 (5%)
**If yes, why** 2			
Routine care	13 (48%)	11 (37%)	24 (42%)
Bleeding	2 (7%)	1 (3%)	3 (5%)
Abdominal pain	2 (7%)	1 (3%)	3 (5%)
Research study	2 (7%)	9 (30%)	11 (19%)
Other/unknown	7 (26%)	6 (20%)	13 (23%)
**If no, why** 3			
Didn’t know what they were/why needed	86 (75%)	186 (78%)	272 (77%)
Heard they were uncomfortable	1 (1%)	1 (0%)	2 (1%)
Couldn’t afford	2 (2%)	4 (2%)	6 (2%)
Didn’t know where to get	3 (3%)	10 (4%)	13 (4%)
Other/unknown	10 (9%)	15 (6%)	25 (7%)
**Seek Pap screening in future**	133 (94%)	247 (92%)	380 (93%)
**If yes, where do you believe you can obtain a Pap smear** 2			
Hospital	114 (86%)	211 (85%)	325 (86%)
Prenatal care	3 (2%)	0 (0%)	3 (1%)
VCT	1 (1%)	2 (1%)	3 (1%)
Family planning clinic	6 (5%)	18 (7%)	24 (6%)
Emergency room	0 (0%)	0 (0%)	0 (0%)
Private doctor	4 (3%)	4 (2%)	8 (2%)
Other	7 (5%)	21 (9%)	28 (7%)
**If yes, how much would you be willing to pay for a Pap smear** 2			
0 Ksh	7 (5%)	14 (6%)	21 (6%)
1 to 400 Ksh	100 (75%)	180 (73%)	280 (74%)
401–999 Ksh	15 (11%)	33 (13%)	48 (13%)
1000+ Ksh	9 (7%)	17 (7%)	26 (7%)
**Prefer at-home self-sampling to assess cancer risk** 5	122 (87%)	221 (82%)	343 (84%)
**Comfortable using a self-sampling device** 5	121 (86%)	216 (81%)	337 (82%)
**Potential concerns regarding self-sampling**			
Proper sample collection	84 (60%)	189 (71%)	273 (67%)
Pain from inserting device	5 (4%)	14 (5%)	19 (5%)
Stretch the vaginal canal	3 (2%)	0 (0%)	3 (1%)
Unable to insert the device into the vagina	6 (4%)	18 (7%)	24 (6%)
Interpreting results	4 (3%)	20 (7%)	24 (6%)
Other^7^	20 (14%)	41 (15%)	61 (15%)
No concerns	23 (16%)	14 (5%)	37 (9%)
**Ever get a vaccine to prevent cervical cancer if no or low cost**	132 (94%)	253 (94%)	385 (94%)

1 Responses for multiple option questions are not mutually exclusive so the total percentage may sum to ≥100%.

2 Denominator includes only the women who responded ‘yes’ to the above question.

3 Denominator includes only the women who responded ‘no’ to the above question.

4 Other includes condom use, douching, birth at older ages, stress, taking antiretroviral treatment.

5 Other includes avoid douching, avoid sexually transmitted infections, avoid condom use.

6 Explanation provided to participants: There is a new method that can be used to assess a women's risk of cervical cancer without going to a medical clinic. It is a vaginal self-sampling device that you would use in your own home. Would you prefer to take a vaginal sample yourself, using a cotton swab similar to a tampon, in the privacy of your home in order to determine if you are at risk of cervical cancer? Would you feel comfortable inserting the swab into your vagina?

7 Other includes: upset partner, beliefs against inserting objects into vagina, side effects from using device, frequency of testing, where to get treatment, device getting stuck in vagina.

Abbreviations: N (number); % (percentage); HIV (human immunodeficiency virus); STI (sexually transmitted infection); HPV (human papillomavirus); IQR (interquartile range); VCT (voluntary counseling and testing center); Ksh (Kenyan shilling).

Very few women (19% HIV-negative and 11% HIV-positive) reported having had a Pap smear prior to the study. Of those who reported having a Pap smear prior to enrollment in the study, most had been done as part of routine care (42%) or as part of a prior research study (19%) and the remaining were conducted for unknown reasons (23%). Those who had never had a Pap smear reported that they did not get screened because they did not know what a Pap smear was or why they needed one (77%). After having at least two Pap smears as part of the study protocol, nearly all women (93%) said that they would seek out Pap screening in the future, with the hospital being the most commonly cited place at which they can be screened. Three-quarters of women said they would be willing to pay up to 400 Kenyan shillings for a Pap smear (approximately 5 US dollars).

When asked about methods of screening and prevention, including self-sampling for HPV testing and HPV vaccination, the majority of the women (82%) reported that they would be comfortable using an at-home cervico-vaginal self-sampling device. In fact, 84% of all women said they would prefer this method over having a sample collected in a clinic. Despite their willingness to consider self-sampling, the majority of women (91%) had concerns regarding self-sampling, with the ability to properly collect the sample being the most commonly cited concern (67%). HIV-negative women were slightly more likely to report no concerns (16%) as compared to HIV-positive women (5%). Nearly all HIV-negative (94%) and HIV-positive women (94%) said they would get a vaccine to prevent cervical cancer if offered to them in the future at no or low cost.

**Figure 1:**
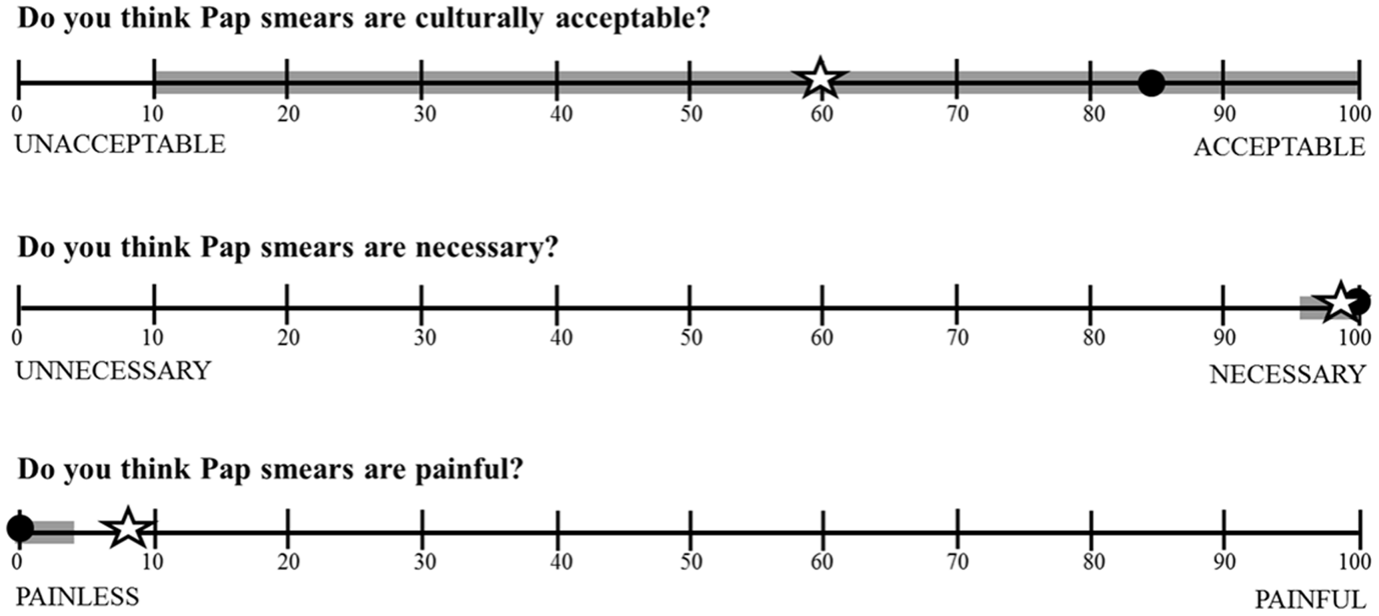
Women’s response regarding their feelings of Pap smear acceptability, feelings of necessity, and how painful they found Pap smears to be over two years of biannual screening. The open star symbol represents mean level of response; the closed circle represents median level of response; the thick gray line represents the interquartile range of responses.

When asked about the level of acceptability, pain, and necessity that they felt towards Pap smear screening, nearly all women (95%) gave Pap smears the highest possible rating for necessity (Figure 1). About half of women (47%) gave Pap smears the highest possible rating for acceptability (100 on a scale from 0 to 100; median = 80 (IQR 10–100)), while 21% felt they were completely unacceptable. When the women, who had undergone biannual screening as part of the study, were asked to rate the level of pain associated with Pap smears, 73% of the women reported the lowest level of pain (0 on a scale from 0 to 100; n = 300) and only 3% (n = 12) gave a rating of ≥50.

### Correlates of Pap Smear, Knowledge and Acceptability

Women age 30 years and older (odds ratio [OR]: 3.6, 95% confidence interval [CI]: 1.9, 7.2), with at least a secondary education (OR = 2.0, 95% CI: 1.1, 3.7), who had ever heard of HPV (OR = 2.2, 95% CI: 1.1, 4.2) or knew that Pap smears were used to prevent invasive cervical cancer (OR = 1.7, 95% CI: 0.8, 3.5) were more likely to have ever had a Pap smear prior to enrolling in the study (Table 3). Women who were HIV-seropositive (OR = 0.5, 95% CI: 0.3, 1.0) or used a condom the last time they had sex prior to baseline (OR = 0.2, 95%CI: 0.1, 0.6) were less likely to have a history of Pap smear screening. Education, knowledge of HPV, HIV-status and condom use remained significantly associated with ever having a previous Pap smear after adjusting for age.

**Table 3:** Association between women’s baseline characteristics and previous pap testing and knowledge and acceptability of Pap screening.

	Ever previous Pap^1^N (%)	OR (95% CI)	Knowledge ofHPV^2^ N (%)	OR (95% CI)	Knowledge ofPap to preventICC^3^ N (%)	OR (95% CI)	Pap screening acceptable^4^N (%)	OR (95% CI)
**Age**								
<30 years	16 (7%)	1 (ref)	39 (18%)	1 (ref)	39 (18%)	1 (ref)	103 (46%)	1 (ref)
≥30 years	41 (22%)	3.6 (1.9, 7.2)	36 (20%)	1.1 (0.7, 1.9)	35 (20%)	1.1 (0.6, 1.9)	90 (48%)	1.1 (0.7, 1.6)
**Married**								
No	0 (0%)	1 (ref)	4 (29%)	1 (ref)	3 (21%)	1 (ref)	6 (43%)	1 (ref)
Yes	57 (15%)	3.3 (0.5, IN)	71 (18%)	0.6 (0.2, 2.5)	71 (18%)	0.8 (0.2, 4.6)	187 (47%)	1.2 (0.4, 4.3)
**Education**								
Primary or below (≤8)	23 (10%)	1 (ref)	24 (11%)	1 (ref)	29 (13%)	1 (ref)	107 (47%)	1 (ref)
Secondary or above (>8)	34 (19%)	2.0 (1.1, 3.7)	51 (28%)	3.2 (1.8, 5.8)	45 (25%)	2.2 (1.3, 3.9)	86 (47%)	1.0 (0.7, 1.5)
**Employed/earning income**								
No	39 (14%)	1 (ref)	50 (18%)	1 (ref)	48 (17%)	1 (ref)	139 (49%)	1 (ref)
Yes	18 (15%)	1.1 (0.5, 2.0)	25 (20%)	1.2 (0.6, 2.0)	26 (21%)	1.3 (0.7, 2.2)	54 (43%)	0.8 (0.5, 1.2)
**History of smoking** 5								
Never	55 (15%)	1 (ref)	69 (18%)	1 (ref)	69 (18%)	1 (ref)	185 (49%)	1 (ref)
Ever	2 (7%)	0.5 (0.1, 1.9)	6 (21%)	1.2 (0.4, 3.1)	5 (17%)	0.9 (0.3, 2.5)	8 (27%)	0.4 (0.1, 0.9)
**Number of live births**								
0 to 1	10 (9%)	1 (ref)	28 (25%)	1 (ref)	29 (26%)	1 (ref)	51 (45%)	1 (ref)
2 to 3	29 (15%)	1.8 (0.8, 4.2)	33 (17%)	0.6 (0.3, 1.1)	33 (17%)	0.6 (0.3, 1.1)	95 (48%)	1.1 (0.7, 1.9)
4+	18 (18%)	2.3 (0.9, 5.8)	14 (15%)	0.5 (0.2, 1.1)	12 (12%)	0.4 (0.2, 0.9)	47 (47%)	1.1 (0.6, 1.9)
**Hormonal contraception** 6								
No	47 (14%)	1 (ref)	58 (18%)	1 (ref)	60 (18%)	1 (ref)	159 (48%)	1 (ref)
Yes	10 (13%)	0.9 (0.4, 1.9)	17 (22%)	1.3 (0.7, 2.5)	14 (18%)	1.0 (0.5, 1.9)	34 (44%)	0.9 (0.5, 1.5)
**Age at sexual debut**								
<18 years	21 (11%)	1 (ref)	30 (18%)	1 (ref)	24 (12%)	1 (ref)	97 (50%)	1 (ref)
≥18 years	36 (17%)	1.6 (0.9, 3.1)	45 (21%)	1.4 (0.8, 2.5)	50 (23%)	2.1 (1.2, 3.7)	96 (44%)	0.8 (0.5, 1.2)
**Lifetime number of sex partners**								
<4	31 (12%)	1 (ref)	50 (19%)	1 (ref)	50 (19%)	1 (ref)	122 (46%)	1 (ref)
≥4	26 (18%)	1.7 (0.9, 3.0)	25 (17%)	0.9 (0.5, 1.5)	24 (17%)	0.8 (0.5, 1.5)	71 (49%)	1.1 (0.7, 1.7)
**Condom use at last sex**								
No	8 (40%)	1 (ref)	1 (5%)	1 (ref)	7 (35%)	1 (ref)	12 (60%)	1 (ref)
Yes	41 (12%)	0.2 (0.1, 0.6)	65 (20%)	4.6 (0.7, 194.4)	62 (18%)	0.4 (0.2, 1.3)	157 (46%)	0.6 (0.2, 1.6)
**HIV-status**								
Seronegative	27 (19%)	1 (ref)	28 (20%)	1 (ref)	28 (20%)	1 (ref)	67 (48%)	1 (ref)
Seropositive	30 (11%)	0.5 (0.3, 1.0)	47 (18%)	0.8 (0.5, 1.5)	46 (17%)	0.8 (0.5, 1.5)	126 (47%)	1.0 (0.6, 1.5)
**Abnormal cytology at baseline**								
No	43 (15%)	1 (ref)	52 (18%)	1 (ref)	57 (19%)	1 (ref)	133 (45%)	1 (ref)
Yes	10 (11%)	0.8 (0.3, 1.6)	18 (21%)	1.2 (0.6, 2.2)	14 (16%)	0.8 (0.4, 1.5)	47 (53%)	1.4 (0.8, 2.3)
**Ever previous Pap**								
No	–	–	58 (17%)	1 (ref)	59 (17%)	1 (ref)	156 (45%)	1 (ref)
Yes	–	–	17 (30%)	2.2 (1.1, 4.2)	15 (26%)	1.7 (0.8, 3.5)	35 (61%)	1.9 (1.1, 3.6)
**Knowledge of HPV**								
No	39 (12%)	1 (ref)	–	–	51 (16%)	1 (ref)	147 (45%)	1 (ref)
Yes	17 (23%)	2.2 (1.1, 4.2)	–	–	23 (31%)	2.4 (1.3, 4.4)	44 (59%)	1.7 (1.0, 3.0)
**Knowledge of Pap to prevent ICC**								
No	42 (13%)	1 (ref)	52 (16%)	1 (ref)	–	–	149 (44%)	1 (ref)
Yes	15 (20%)	1.7 (0.8, 3.5)	23 (31%)	2.4 (1.3, 4.4)	–	–	44 (59%)	1.8 (1.1, 3.2)

1 Compares women who reported Pap smear screening prior to enrollment in the study (n = 57) to women with no previous screening (n = 347).

2 Compares women who had heard of HPV (n = 75) to the women who had never heard of HPV (n = 328).

3 Compares women who know (n = 74) and do not know (n = 335) that Pap smear screening is used to prevent invasive cervical cancer.

4 Compares women who gave Pap smear screening the highest rating (100 on scale from 0 to 100) for acceptability (n = 193) to those who gave less than the highest (<100) acceptability rating (n = 216).

5 History of smoking: ≥1 cig/day for ≥6 consecutive months.

6 Hormonal contraception includes injection, oral and implant-based contraceptive.

Abbreviations: OR (unadjusted odds ratio); CI (confidence interval); N (number); % (percentage); HIV (human immunodeficiency virus); HPV (human papillomavirus); IQR (interquartile range); ICC (invasive cervical cancer); IN (infinity, could not reliably estimate because of small numbers).

Missing data: Hormonal contraception (n = 1); Median age at sexual debut (n = 1); Lifetime number of partners (n = 1); Condom use at last sex (n = 51); abnormal cytology at baseline (n = 25).

Women with at least a secondary education compared to less than a secondary education (OR = 3.2, 95%CI: 1.8, 5.8), those who had ever had a Pap smear prior to enrollment in the study compared to never previously screened (OR = 2.2, 95%CI: 1.1, 4.2), and those who knew compared with those who did not know that Pap smears were conducted to as part of preventing cervical cancer (OR = 2.4, 95%CI: 1.3, 4.4) were more likely to have heard of HPV. Similarly, women with at least a secondary education compared to less than a secondary education (OR = 2.2, 95%CI: 1.3, 3.9) and those who had ever versus never heard of HPV (OR = 2.4, 95%CI: 1.3, 4.4) were more likely to know that Pap smears can identify cervical abnormalities before cancer develops in order to prevent the development of invasive cancer. A woman’s history of Pap smear screening (OR = 1.9, 95%CI: 1.1, 3.6), knowledge of HPV (OR = 1.7, 95%CI: 1.0, 3.0) and knowledge that Pap smears are a tool to prevent invasive cervical cancer (OR = 1.8, 95%CI: 1.1, 3.2) were associated with giving routine Pap smear screening the highest possible rating for acceptability. Although education was associated with a history of Pap smear screening, knowledge of HPV and knowledge that Pap smears are used in the prevention of cervical cancer, education was not associated with acceptability of Pap smears (OR = 1.0, 95% CI: 0.7, 1.5).

## Discussion

The majority of women knew that Pap smears are used to detect cervical cancer (69%), but very few knew that routine Pap screening is the main way to prevent cervical cancer (18%). Most women did not know the cause of cervical cancer (78%) and only 18% had ever heard of HPV. However, it was reassuring to find that most women held positive attitudes towards future Pap smear screening, self-sampling, and HPV vaccination. Our findings highlight the need to educate and reinforce to women that routine Pap smear screening is a key part of preventing invasive cervical cancer so that the fear of being diagnosed with cancer is not a barrier to screening.

Despite the fact that knowledge of HPV was very low in our cohort, almost all women reported that they would be willing to be vaccinated against HPV if the vaccine was available at no or low cost. This finding is similar to other studies [28], [29], including one from Kenya which found that 95% of women would likely vaccinate their daughters to prevent cervical cancer [9]. However, this previous comprehensive vaccine assessment study found that only 31% of those women still said they would vaccinate their daughter if it took three injections and 75% of women said they would only pay 100 Kenyan shillings (approximately 1.25 United States Dollars [USD]) out of pocket to vaccinate their daughter. Current generation prophylactic HPV vaccines cost approximately 375 USD in developed countries and require three injections over a 6 month period. However, the GAVI Alliance now includes the HPV vaccine on its list of childhood vaccines available for funding in resource-limited countries. Also, studies are currently underway to assess the efficacy of a shortened 1 or 2 dose vaccine administration schedule.

Consistent with previous studies that document low uptake of Pap smear screening in sub-Saharan Africa, previous Pap smear screening was low in our cohort, with most women (86%) reporting never having had a Pap smear prior to joining the study [30], [31]. It was interesting to find that even though HIV-positive women are generally more likely to be in contact with the healthcare system, and despite the recognized link between HIV and cervical precancer [32], [33] and the recommendation for women to have biannual screening during the first year after HIV diagnosis, HIV-positive women were less likely to have had a previous Pap smear compared to HIV-negative women. This highlights the importance of integrating cervical cancer screening and management with routine HIV care and treatment programs, as suggested by the 2011 national guidelines for antiretroviral therapy in Kenya [34].

Without an intervention or educational campaign, it is likely that the strongest predictor of future screening is having been screened in the past. Identifying the characteristics of women who have and have not had previous screening can help to target screening and outreach efforts. In this cohort, women with less education, who were HIV-positive, or didn’t have knowledge of HPV or Pap smears, were less likely to have had previous Pap smear screening. Given the cross-sectional study design, it is unclear whether women learned of HPV during Pap smear screening or whether they learned about HPV from other sources and thus sought out cervical cancer screening. However, in a setting such as Nairobi, where Pap smear screening is available, although access and campaigns are limited, and where HPV testing is now being advertised, it is likely that women who had previous Pap smears for a research study, routine care or diagnostic purposes learned of HPV during or in response to screening. In addition, these findings are consistent with a previous study of cervical cancer patients and non-patients in Nairobi, which found a lower level of education and lack of knowledge of cervical cancer to be associated with a decreased likelihood of having had previous pap screening [9]. Previous studies have also shown that knowledge of cervical cancer and Pap smears can influence the uptake of cervical cancer screening services [35], [36]. In our study, women who had heard of HPV and knew that Pap smears are used to prevent invasive cervical cancer were far more likely to find Pap screening 100% acceptable and necessary as compared to women without knowledge of HPV and the role of Pap smears in cervical cancer prevention.

Women’s knowledge and attitudes were self-reported when they exited the study so there was no follow-up to confirm their feelings or intentions. Pap smear screening conducted during the study may have influenced women’s perception towards Pap smear acceptability, as compared to women who have never been screened. However, after having at least one Pap smear during the study, women reported little physical discomfort associated with Pap smear screening and most responded positively toward future use of a self-sampling device and HPV vaccination. All women in our cohort were in stable, HIV-discordant partnerships and so their responses may not be generalizable to other HIV-negative and HIV-positive women in the general population. However, this is a very relevant population of women since they are of screening age, HIV-positive or at risk for HIV, and many are mothers with daughters potentially eligible for HPV prophylactic vaccination. Furthermore, very little data exist on women’s knowledge and attitudes towards cervical cancer and prevention, especially from East Africa where the incidence and mortality rate of cervical cancer are one of the highest worldwide [1]. Our ability to examine within one population attitudes towards both traditional screening methods, such as Pap smears, and towards new alternative methods, such as the use of self-sampling and HPV vaccination, does differentiate this study from previous surveys by providing new data on acceptability and correlates of several modalities of cervical cancer prevention. These data are an important first step to developing and successfully implementing effective and acceptable screening programs. Community-based assessment of knowledge and acceptability, including women who have never had Pap smear screening and women who have used self-sampling devices, is an important next step to obtain information that reflects the diversity of the target population.

Despite low levels of knowledge of HPV and that the fact that Pap smears are a tool to prevent, not just detect, cervical cancer, women reported a high-level of acceptability for cervical cancer prevention measures, including Pap screening, HPV vaccination, and self-sampling. Our findings highlight the need for education regarding the cause and prevention of cervical cancer. Currently over 100 sites in Kenya offer regular screening, however, awareness is low and cervical cancer screening coverage for all women age 18–69 years is only 3.2% [5]. Therefore, start-up of a successful screening program requires bringing the resources into the community but also mobilizing the unscreened population to participate in the program. Data from this study can help inform educational and outreach programs to target high-risk women, with the goal of eliminating cervical cancer worldwide through the use of various screening and prevention tools.
